# Skeletal Muscle Pyruvate Dehydrogenase Phosphorylation and Lactate Accumulation During Sprint Exercise in Normoxia and Severe Acute Hypoxia: Effects of Antioxidants

**DOI:** 10.3389/fphys.2018.00188

**Published:** 2018-03-19

**Authors:** David Morales-Alamo, Borja Guerra, Alfredo Santana, Marcos Martin-Rincon, Miriam Gelabert-Rebato, Cecilia Dorado, José A. L. Calbet

**Affiliations:** ^1^Department of Physical Education, University of Las Palmas de Gran Canaria, Las Palmas de Gran Canaria, Spain; ^2^Research Institute of Biomedical and Health Sciences, Las Palmas de Gran Canaria, Spain; ^3^Clinical Genetics Unit, Complejo Hospitalario Universitario Insular-Materno Infantil de Las Palmas de Gran Canaria, Las Palmas de Gran Canaria, Spain

**Keywords:** sprint exercise, skeletal muscle, hypoxia, human, oxidative stress, pyruvate dehydrogenase, PDH

## Abstract

Compared to normoxia, during sprint exercise in severe acute hypoxia the glycolytic rate is increased leading to greater lactate accumulation, acidification, and oxidative stress. To determine the role played by pyruvate dehydrogenase (PDH) activation and reactive nitrogen and oxygen species (RNOS) in muscle lactate accumulation, nine volunteers performed a single 30-s sprint (Wingate test) on four occasions: two after the ingestion of placebo and another two following the intake of antioxidants, while breathing either hypoxic gas (P_I_O_2_ = 75 mmHg) or room air (P_I_O_2_ = 143 mmHg). *Vastus lateralis* muscle biopsies were obtained before, immediately after, 30 and 120 min post-sprint. Antioxidants reduced the glycolytic rate without altering performance or VO_2_. Immediately after the sprints, Ser^293^- and Ser^300^-PDH-E1α phosphorylations were reduced to similar levels in all conditions (~66 and 91%, respectively). However, 30 min into recovery Ser^293^-PDH-E1α phosphorylation reached pre-exercise values while Ser^300^-PDH-E1α was still reduced by 44%. Thirty minutes after the sprint Ser^293^-PDH-E1α phosphorylation was greater with antioxidants, resulting in 74% higher muscle lactate concentration. Changes in Ser^293^ and Ser^300^-PDH-E1α phosphorylation from pre to immediately after the sprints were linearly related after placebo (*r* = 0.74, *P* < 0.001; *n* = 18), but not after antioxidants ingestion (*r* = 0.35, *P* = 0.15). In summary, lactate accumulation during sprint exercise in severe acute hypoxia is not caused by a reduced activation of the PDH. The ingestion of antioxidants is associated with increased PDH re-phosphorylation and slower elimination of muscle lactate during the recovery period. Ser^293^ re-phosphorylates at a faster rate than Ser^300^-PDH-E1α during the recovery period, suggesting slightly different regulatory mechanisms.

## Introduction

During sprint exercise in normoxia lactate accumulates in the recruited skeletal muscles (Jones et al., 1985; Cheetham et al., 1986; Parolin et al., 1999; Morales-Alamo et al., 2012), indicating that the flux through the pyruvate dehydrogenase (PDH) is not sufficient to avoid pyruvate accumulation and its corresponding reduction to lactate (Howlett et al., 1999). Compared to sprint exercise in normoxia, sprint exercise in severe acute hypoxia is accompanied by increased lactate accumulation (McLellan et al., 1990; Morales-Alamo et al., 2012) and greater reactive nitrogen and oxygen species (RNOS) production and more oxidative stress (Cuevas et al., 2005; Morales-Alamo et al., 2012; Morales-Alamo and Calbet, 2014). Animal experiments have shown that excessive RNOS production may reduce PDH activity (Churchill et al., 2005). It remains unknown whether the increased lactate production during sprint exercise in severe acute hypoxia is due, at least in part, to reduced activation of PDH.

Putman et al. (1995) showed that during repeated 30-s isokinetic sprints in normoxia lactate production was not dependent on O_2_ delivery. However, we have recently shown that during the last 15 s of a 30-s isokinetic sprint in severe acute hypoxia skeletal muscle VO_2_ is indeed limited by O_2_ delivery (Calbet et al., 2015). Moreover, during submaximal exercise at 55% of the VO_2_max in acute hypoxia (F_i_O_2_: = 0.11), the level of PDH activation after 1 min cycling was lower than in normoxia (Parolin et al., 2000).

**Figure 1:**
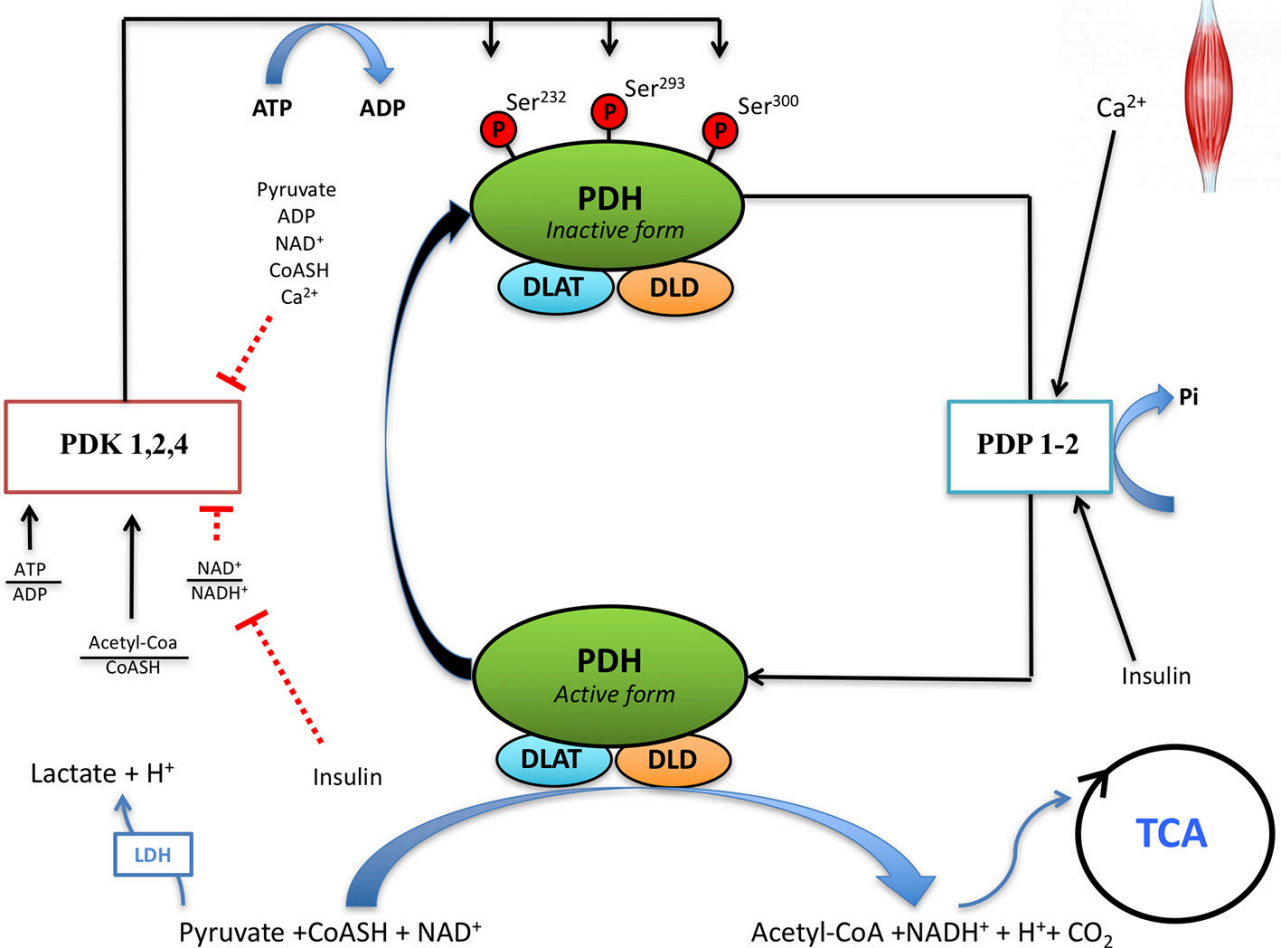
Regulation of pyruvate dehydrogenase (PDH) activity in skeletal muscle. Pyruvate dehydrogenase complex (PDC) is regulated by phosphorylation and dephosphorylation by pyruvate dehydrogenase kinase (PDK) and pyruvate dehydrogenase phosphatase (PDP), respectively. PDC catalyzes the irreversible decarboxylation of pyruvate to Acetyl-CoA, which can be oxidized through the tricarboxylic acid cycle (TCA). DLAT, dihydrolipoamide S-acetyltransferase; DLD, dihydrolipoamide dehydrogenase; ATP, adenosine triphosphate; ADP, adenosine diphosphate; NAD, nicotinamide adenine dinucleotide; NADH, nicotinamide adenine dinucleotide reduced; CoASH, coenzyme A.

Increased acidification during exercise in hypoxia may reduce nicotinamide adenine dinucleotide phosphate-oxidase 2 (NOX2) activity, which is considered the main source of ion superoxide production during exercise (Simchowitz, 1985; Morgan et al., 2005; Brennan-Minnella et al., 2015), particularly during brief periods (10–15 min) of contractile activity (Jackson, 2015). Superoxide stimulates Ca^2+^ efflux through the ryanodine receptors of the sarcoendoplasmic reticulum (Jackson et al., 2007; Dulhunty et al., 2017). The increased (Ca^2+^) in the sarcoplasm is necessary for the activation of PDH phosphatases, which dephosphorylate and activate PDH (Hucho et al., 1972; Wieland, 1983; Behal et al., 1993) (Figure 1). Thus, the increased muscle acidification during sprint exercise in hypoxia may reduce PDH activation by at least four mechanisms: (1) by decreasing Ca^2+^ efflux from the sarcoplasmic reticulum (Bailey et al., 2007; Dulhunty et al., 2017), (2) by inducing the activation of pyruvate dehydrogenase kinase (PDK) (Churchill et al., 2005; Wu et al., 2013), (3) by causing oxidative damage to the enzyme (Tabatabaie et al., 1996), and (4) by reducing PDH phosphatase activity (Radak et al., 2013). In the case of an inhibitory influence of exercise-induced RNOS on PDH activation, the ingestion of antioxidants before sprint exercise should enhance PDH dephosphorylation and activation, leading to lower accumulation of lactate.

Pyruvate dehydrogenase is activated by dephosphorylation of serine residues (Ser^232^, Ser^293^, Ser^300^) located in the E1α subunit of PDH (Yeaman et al., 1978) (Figure 1). The balance between the activities of pyruvate dehydrogenase kinases (PDKs) and PDH phosphatases (PDP) determines the degree of PDH activation (Hucho et al., 1972; Wieland, 1983; Behal et al., 1993).

Therefore, the main aim of this study was to determine whether PDH activation, as determined by the assessment of its phosphorylation level, is reduced during sprint exercise in hypoxia compared to the same exercise performed in normoxia. Another aim was to ascertain whether antioxidants administration immediately before a sprint exercise in normoxia or hypoxia influences PDH activity during the subsequent recovery period.

## Materials and methods

### Subjects

Initially, 10 healthy male physical education students agreed to participate in this investigation, but completed data for all conditions were obtained in nine of them (age = 25 ± 5 year, height = 176.0 ± 5.1 cm, body mass = 79.4 ± 10.1 kg, body fat = 18.3 ± 6.7%, Table 1). The study was performed in accordance with the Declaration of Helsinki and was approved by the Ethical Committee of the University of Las Palmas de Gran Canaria (CEIH-2010-01). All subjects signed a written informed consent before entering the study.

**Table 1:** Physical characteristics and ergoespirometric variables during incremental and sprint exercise in normoxia and severe acute hypoxia (mean ± *SD*).

	**Normoxia**	**Hypoxia**
Age (years)	25.2 ± 4.7	
Height (cm)	176.0 ± 5.1	
Weight (Kg)	80.2 ± 9.9	
% body fat	18.3 ± 6.7	
Two-Legs lean mass (Kg)	19.5 ± 2.4	
SHRpeak (Beats/min)	175.2 ± 8.0	173.5 ± 9.2^*^
VO_2_max (L/min)	3.850 ± 0.308	2.586 ± 0.208^*^
Wmax (W)	356.3 ± 38.62	285.3 ± 32.1^*^
Wpeak (W)	999.11 ± 129.0	944.3 ± 131.3
Wpeak/Kg LLM (W/kg)	16.6 ± 2.0	15.6 ± 1.4
Wmean (W)	575.3 ± 60.7	539.8 ± 69.0^*^
Wmean/Kg LLM (W/kg)	9.56 ± 0.85	8.95 ± 0.78^*^
O_2_ Demand (L/min)	8.390 ± 0.798	7.830 ± 0.765^*^
Accumulated VO_2_ (L)	1.192 ± 0.406	0.847 ± 0.123^*^
O_2_ deficit (L)	3.003 ± 0.498	3.068 ± 0.399
O_2_ deficit/Wmean	5.24 ± 0.83	5.70 ± 0.45^*^
Wingate SpO_2_	98.7 ± 0.6	80.6 ± 4.4^*^
Wingate P_ET_O_2_	114.1 ± 6.9	48.8 ± 3.3^*^

*SHRpeak, peak heart rate during the sprints; Wmax, maximal intensity during the incremental exercise test to exhaustion; Wpeak, peak power output during the Wingate test; LLM, lean mass of the lower extremities; Wmean, mean power output during the Wingate test; Accumulated VO_2_, amount of O_2_ consumed during the 30 s Wingate test; SpO_2_, Hemoglobin saturation (pulse oximetry); P_ET_O_2_, end tidal O_2_ pressure*.

* *P < 0.05 compared to normoxia*.

### General procedures

Body composition was determined by dual-energy x-ray absorptiometry (Hologic QDR-1500, Hologic Corp., software version 7.10, Waltham, MA) as described elsewhere (Calbet et al., 1998). After familiarization, subjects reported to the laboratory to complete different tests on separate days. First their peak VO_2_ (VO_2_peak), maximal heart rate (HRmax) and maximal power output (Wmax) were determined in normoxia (F_I_O_2_: 0.21, P_I_O_2_: 143 mmHg) and severe acute hypoxia (F_I_O_2_: 0.105, P_I_O_2_: 75 mmHg) with an incremental exercise test to exhaustion (50 W/min), as previously described (Morales-Alamo et al., 2012, 2013, 2017). Hemoglobin oxygen saturation (SpO_2_) was determined with a finger pulse oximeter (OEMIII module, 4549-000, Plymouth, MN). The main experiments consisted on a single 30-s isokinetic sprint at 100 revolutions per minute (Wingate Test) repeated on 4 different days, at least 1 week apart. The Wingate tests were carried out in normoxia (F_I_O_2_: 0.21, P_I_O_2_: 143 mmHg) and acute hypoxia (F_I_O_2_: 0.105, P_I_O_2_: 75 mmHg), both preceded by the ingestion of either placebo (200 mg of starch from corn, Sigma-Aldrich, ref. 14302) or antioxidants, with a double-blind design. Both the placebo and the antioxidants were introduced in microcrystalline cellulose capsules of identical characteristics.

Antioxidant supplements were administered in two doses separated by 30 min, with the first dose ingested 2 h before the sprint exercise, as previously reported (Morales-Alamo et al., 2017). The first dose contained 300 mg of α-lipoic acid, 500 mg vitamin C, and 200 IU vitamin E, whereas the second included 300 mg α-lipoic acid, 500 mg vitamin C, and 400 IU vitamin E (water dispersible). This antioxidant cocktail effectively decreases free radicals levels in blood as demonstrated by paramagnetic spin spectroscopy in humans (Richardson et al., 2007). We assume that similar effects were achieved in skeletal muscle as reflected by two facts. Firstly, these antioxidants blunted the RNOS-mediated signaling response elicited by sprint exercise and, secondly, attenuated the formation of protein carbonyls in this study, as previously reported (Morales-Alamo et al., 2013, 2017).

The experimental days subjects reported to the laboratory early in the morning after a 12 h overnight fast. Then, an antecubital vein was catheterized and after 10 min of rest in the supine position a 20 ml blood sample was withdrawn and used to measure serum glucose and insulin. Right after, a muscle biopsy was obtained from the middle portion of the *Vastus lateralis* muscle using the Bergstrom's technique with suction (Guerra et al., 2011). After the resting muscle biopsy, the subjects sat on the cycle ergometer for 4 min while breathing either room air or a hypoxic gas mixture from a gas cylinder.

Within 10 s following the sprint, a second muscle biopsy was taken, and then another blood sample was obtained. During the following 2 h, the subjects had free access to water and sat quietly in the laboratory while two additional muscle biopsies and blood samples were obtained at 30 and 120 min post-Wingate. For the last two biopsies, a new incision was performed in the contralateral leg. To avoid injury-triggered activation of signaling cascades, the muscle biopsies were obtained at least 3 cm apart, using the procedures described by Guerra et al. (2011). The muscle specimens were fast cleaned to remove any visible blood, fat, or connective tissue. Then the muscle tissue was immediately frozen in liquid nitrogen and stored at −80°C for later analysis.

### Oxygen uptake

Oxygen uptake was measured with a metabolic cart (Vmax N29; Sensormedics, Yorba Linda, California, USA), as previously reported (Morales-Alamo et al., 2012, 2013, 2017). Respiratory variables were analyzed breath-by-breath and averaged every 5 s during the Wingate test and every 20 s during the incremental exercise tests (Calbet et al., 1997). The highest 20-s averaged VO_2_ recorded in normoxia and hypoxia was taken as the normoxic and hypoxic VO_2_peak values, respectively.

### Muscle metabolites

From each muscle biopsy, 30 mg of wet tissue were treated with 0.5 M HClO_4_ and centrifuged at 15,000 g at 4°C for 15 min. The supernatant was neutralized with KHCO_3_ 2.1M. Phosphocreatine (PCr), creatine (Cr), muscle glucose (GLU), glucose-6-phosphate (G6P), glucose-1-phosphate (G1P), fructose-6-phosphate (F6P), pyruvate (Pyr), and lactate (Lac) were enzymatically determined in neutralized extracts by fluorometric analysis (Lowry and Passonneau, 1972; Gorostiaga et al., 2010). Alanine (Ala) muscle accumulation was assumed to be equivalent to 1.43-fold that of pyruvate accumulation (Katz et al., 1986). Muscle metabolite concentrations were adjusted to the individual mean total creatine (PCr + Cr) because this mean should remain constant during the exercise (Harris et al., 1976). The adjustment to total creatine content accounts for variability in solid non-muscle constituents, which may be present in the biopsies (Parra et al., 2000). This correction was applied to all metabolites including lactate. This assumes that the increase in interstitial lactate and intracellular water was similar in the four trials and that the majority of the lactate produced during the sprints was retained inside the muscle fibers (Calbet et al., 2003). The glycolytic rate (GR) was calculated as GR = 0.5 × (Δ Lac + Δ Pyr + Δ Ala), and glycogenolytic rate (GGR) as GGR = Δ G1P + Δ G6P + Δ F6P + 0.5 × (Δ Lac + Δ Pyr + Δ Ala) (Chasiotis et al., 1982). The NAD^+^/NADH^+^ and ATP/ADP ratios were calculated as previously reported (Morales-Alamo et al., 2017).

### Total protein extraction, electrophoresis, and western blot analysis

Muscle protein extracts were prepared as described previously (Guerra et al., 2007) with Complete protease inhibitor cocktail and PhosSTOP phosphatase inhibitor cocktail tablets (Roche Diagnostics, Mannheim, Germany). Total protein content was quantified using the bicinchoninic acid assay (Smith et al., 1985). Fifty microgram of each sample were subjected to immunobloting (Guerra et al., 2010) using Immun-BlotTM PVDF membranes from Bio-Rad Laboratories (Hemel Hempstead Hertfordshire, UK). Ser^293^-PDH-E1α and Ser^300^-PDH-E1α phosphorylation and total PDH were determined by western blot (50 μg in each lane) using specific antibodies (AP1062 and AP1064 from Calbiochem, Darmstadt, Germany; and sc-133898, Santa Cruz Biotechnology, CA, USA). Antibodies were diluted in 4% bovine serum albumin in Tris-buffered saline with 0.1% Tween 20 (TBS-T) (BSA-blocking buffer). To control for differences in loading and transfer efficiency, membranes were incubated with a monoclonal mouse anti-α-tubulin antibody (T-5168-ML, Sigma, St. Louis, MO, USA) diluted in TBS-T with 5% blotting grade blocker non-fat dry milk (blotto-blocking buffer). No significant changes were observed in α-tubulin protein levels during the experiments. Antibody-specific labeling was revealed by incubation with a HRP-conjugated goat anti-rabbit antibody (1:20,000; 111-035-144, Jackson ImmunoResearch, Baltimore, USA) antibody, both diluted in 5% blotto blocking buffer and specific bands were visualized with the Inmmun-Star™ WesternC™ kit, using the ChemiDoc XRS system (Bio-Rad Laboratories, Hercules, CA, USA) and analyzed with the image analysis program Quantity one© (Bio-Rad laboratories, Hercules, CA, USA). The densitometry analysis was carried out immediately before saturation of the immunosignal. Muscle-signaling data were represented as a percentage of immunostaining values obtained for the phosphorylated form of each kinase relative to the respective total form. Samples from each subject trial were run on the same gel. In addition, in all gels a human muscle sample obtained from a healthy young man was used as an internal control, to reduce inter-gel variability (Bass et al., 2017).

### Statistics

Variables were checked for normal distribution by using the Shapiro-Wilks test. A three-way repeated-measures ANOVA was used with three within subject factors: F_I_O_2_ (with two levels: normoxia and hypoxia), antioxidants (with two levels: antioxidants and placebo), and time (with four levels: pre-exercise, end-exercise, 30 and 120 min recovery). The Mauchly's test of sphericity was run before the ANOVA, and in the case of violation of the sphericity assumption, the degrees of freedom were adjusted according to the Huynh and Feldt test. When a significant main effect or interaction was observed, specific pairwise comparisons were carried out with the least significant difference *post-hoc* test. The relationship between variables was determined using linear regression analysis. The areas under the curve (AUC) were determined using the trapezoidal rule and compared between conditions with paired Student *t*-tests. Values are reported as the mean ± standard deviation of the mean (unless otherwise stated). *P* ≤ 0.05 was considered significant. Statistical analysis was performed using SPSS v.15.0 for Windows (SPSS Inc., Chicago, IL).

## Results

### Performance and metabolism

Part of the effects on performance (Table 1) and muscle metabolites (Table 2) have been published previously (Morales-Alamo et al., 2012, 2013, 2017) and are only summarized here. Peak power output was similar between conditions. Mean power output was reduced by 5.3% in hypoxia (*P* = 0.03) and was not affected by the ingestion of antioxidants (558 ± 62 and 559 ± 61 W, placebo and antioxidants, respectively *P* = 0.81). The accumulated VO_2_ during the Wingate test was reduced by 30% in hypoxia (*P* = 0.006) and was not altered by the ingestion of antioxidants (1.095 ± 0.242 and 1.098 ± 0.267 L, placebo and antioxidants, respectively *P* = 0.99). The mean muscle lactate concentration at the end of the sprint was 23% lower after the ingestion of antioxidants (46.8 ± 13.4 and 36.2 ± 12.1 mmol.kg wet^−1^, mean of the two placebo and mean of the two antioxidants conditions, respectively, *P* = 0.004) and 30% higher in hypoxia than normoxia (46.9 ± 13.9 and 36.1 ± 12.2 mmol.kg wet^−1^, mean of the two hypoxia and the two normoxia conditions, respectively, *P* = 0.004). The ingestion of antioxidants reduced the glycolytic rate by 25% from 22.3 ± 6.8 to 16.9 ± 6.0 mmol.kg^−1^. (30 s)^−1^ (*P* = 0.002), while the reduction in the glycogenolytic rate did not reach statistical significance (from 27.2 ± 7.0 to 25.4 ± 10.3 mmol.kg^−1^. (30 s)^−1^, for the mean of the two placebo and the two antioxidants conditions, respectively, *P* = 0.49). Compared to placebo, 30 min after the end of the sprints preceded by antioxidants muscle lactate concentration was 74% greater (4.3 ± 3.0 vs. 8.1 ± 4.5 mmol.kg wet^−1^, mean of the two placebo and the two antioxidants conditions, respectively, *P* = 0.02). The ratio ATP/ADP was similarly reduced after the sprints and recovered to pre-exercise levels 30 min later, regardless of the F_I_O_2_ and antioxidants ingestion (Table 2). Immediately after the sprints, the NAD^+^/NADH^+^ ratio was decreased more in hypoxia than normoxia (from 618 ± 355 to 42.5 ± 14.5 and from 452 ± 201 to 72.8 ± 28.6, mean of the two hypoxia and the two normoxia conditions, respectively, *P* = 0.003). The NAD^+^/NADH^+^ ratio was lower after the administration of antioxidants (*P* = 0.03, antioxidants main effect) (Table 2).

**Table 2:** Muscle metabolites before, immediately after, and 30 min following a 30-s all-out sprint (Wingate test) in normoxia (P_I_O_2_ = 143 mmHg) and hypoxia (P_I_O_2_ = 74 mmHg), after the ingestion of antioxidants or placebo.

	**Placebo**		**Antioxidants**		**Time**	**Antiox**	**Antiox × Time**	**F_I_O_2_**	**F_I_O_2_ × Time**	**Antiox × F_I_O_2_ × Time**
	**Hypoxia**	**Normoxia**	**Hypoxia**	**Normoxia**						
Pre PCr	16.5 ± 1.5	16.0 ± 2.8	18.2 ± 2.3	17.8 ± 2.3	<0.001	0.12	0.78	0.49	0.75	0.97
Post PCr	6.0 ± 3.3^a^	5.0 ± 2.2^a^	6.8 ± 3.1^a^	6.0 ± 2.9^a^						
Post 30 min PCr	18.8 ± 4.0	18.4 ± 1.9	19.3 ± 2.0	19.6 ± 2.3						
Pre Cr	11.9 ± 1.5	12.4 ± 2.8	10.2 ± 2.3	10.6 ± 2.3	<0.001	0.12	0.78	0.49	0.75	0.97
Post Cr	22.4 ± 3.3^a^	23.5 ± 2.2^a^	21.6 ± 3.1^a^	22.4 ± 2.9^a^						
Post 30 min Cr	9.6 ± 4.0	10.1 ± 1.9	9.1 ± 2.0	8.8 ± 2.3						
Pre ATP	4.8 ± 1.0	5.1 ± 1.9	5.2 ± 1.2	5.0 ± 1.6	<0.001	0.41	0.009	0.9	0.016	0.37
Post ATP	3.2 ± 1.6^a^	2.4 ± 0.8^a^	3.1 ± 0.8^a^	3.5 ± 1.4^a^						
Post 30 min ATP	4.6 ± 2.0	3.5 ± 1.4	3.5 ± 1.1	4.8 ± 1.1						
Pre ADP	0.4 ± 0.2	0.5 ± 0.3	0.3 ± 0.2	0.3 ± 0.2	<0.001	0.4	0.85	0.074	0.76	0.047
Post ADP	0.9 ± 0.8	0.7 ± 0.5	0.4 ± 0.2	0.9 ± 0.8^a^						
Post 30 min ADP	0.3 ± 0.2	0.2 ± 0.1	0.2 ± 0.1	0.2 ± 0.2						
Pre ATP/ADP	13.6 ± 3.2	13.3 ± 5.6	18.3 ± 6.3	17.3 ± 6.8	<0.001	0.28	0.52	0.33	0.64	0.49
Post ATP/ADP	5.9 ± 3.8^a^	4.5 ± 2.5^a^	7.9 ± 2.9^a^^b^	6.0 ± 4.3^a^						
Post 30 min ATP/ADP	27.2 ± 25.2	18.3 ± 5.3	21.8 ± 7.9	22.8 ± 8.0						
Pre G1P	0.04 ± 0.05	0.02 ± 0.02	0.05 ± 0.04	0.06 ± 0.07	0.001	0.18	0.14	0.901	0.17	0.07
Post G1P	0.25 ± 0.30^a^	0.11 ± 0.10^a^	0.34 ± 0.21^a^	0.38 ± 0.32^a^						
Post 30 min G1P	0.05 ± 0.03	0.15 ± 0.16	0.10 ± 0.13	0.11 ± 0.12						
Pre G6P	0.50 ± 0.33	0.47 ± 0.38	0.46 ± 0.31	0.32 ± 0.37	<0.001	0.21	0.22	0.787	0.01	0.08
Post G6P	4.40 ± 3.21^a^	5.38 ± 1.97^a^	10.34 ± 6.98^a^	5.88 ± 4.77^a^						
Post 30 min G6P	1.21 ± 1.60	2.96 ± 6.37	1.44 ± 1.92	4.39 ± 5.29						
Pre F6P	0.08 ± 0.06	0.05 ± 0.06	0.06 ± 0.07	0.06 ± 0.06	0.005	0.06	0.2	0.098	0.34	0.96
Post F6P	0.24 ± 0.30	0.36 ± 0.32^a^	0.50 ± 0.41^a^	0.59 ± 0.42^a^						
Post 30min F6P	0.09 ± 0.11	0.22 ± 0.55	0.24 ± 0.32	0.59 ± 0.40						
Pre Pyr	0.14 ± 0.05	0.08 ± 0.04	0.09 ± 0.04	0.10 ± 0.07	0.02	0.16	0.08	0.09	0.11	0.11
Post Pyr	0.23 ± 0.07^a^	0.26 ± 0.13^a^	0.17 ± 0.07	0.27 ± 0.09^a^						
Post 30 min Pyr	0.07 ± 0.06	0.16 ± 0.10^a^	0.10 ± 0.09	0.13 ± 0.10						
Pre Lac	2.1 ± 1.3	2.6 ± 2.0	3.1 ± 2.0	2.4 ± 2.2	<0.001	0.06	0.002	0.009	0.002	0.26
Post Lac	55.4 ± 20.1^a^^,^^b^^,^^c^	38.2 ± 12.3^a^	38.3 ± 10.6^a^^,^^c^	34.1 ± 14.9^a^						
Post 30 min Lac	3.7 ± 2.9	5.0 ± 3.2	7.3 ± 8.8	8.9 ± 4.9^a^						
Pre NAD^+^/NADH^+^ × 10^−7^	897 ± 667	405 ± 250	339 ± 262	501 ± 363	<0.001	0.03	0.23	0.21	0.3	0.018
Post NAD^+^/NADH^+^ × 10^−7^	43 ± 21^a^	60 ± 22^a^	42 ± 16^a^^,^^b^^,^^c^	85 ± 51^a^^,^^d^						
Post 30 min NAD^+^/NADH^+^ × 10^−7^	256 ± 232^a^	350 ± 313	348 ± 421	153 ± 120^a^						

*Values are means ± SD. Concentrations are reported in mmol.kg^−1^ muscle wet weight*.

a *P < 0.05 Compared with Pre sprint*.

b *P < 0.05 compared with normoxia placebo at the same time point*.

c *P < 0.05 compared with normoxia antioxidants at the same time point*.

d *P < 0.05 compared with hypoxia placebo at the same time point. Pre, Pre sprint; Post, Post sprint; Post 30 min, 30 min into the recovery period; Antiox, antioxidants; Lac, lactate; Pyr, Pyruvate. n = 9*.

**Figure 2:**
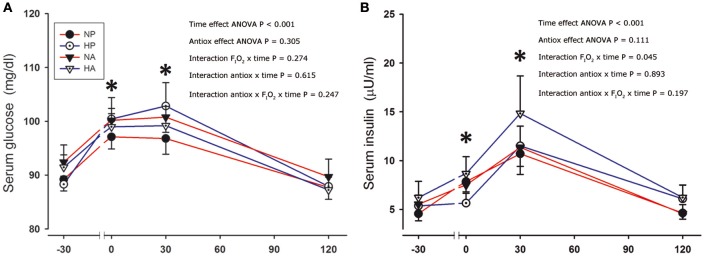
Serum glucose and insulin. Serum glucose **(A)** and insulin concentration **(B)** before, immediately after, and 30 and 120 min after the end of a single 30-s all-out sprint (Wingate test) performed in normoxia placebo (black circles), hypoxia placebo (open circles, F_I_O_2_:0.105), normoxia antioxidants (black triangles) and hypoxia antioxidants (open triangles; F_I_O_2_:0.105). ^*^P < 0.05 in comparison to resting (ANOVA, time main effect, *n* = 9).

Serum glucose and insulin concentrations were increased after the sprints (Figure 2). Although the changes in serum glucose concentration were similar after the four conditions, the serum insulin concentration tended to be marginally greater (+15%) after the sprints preceded by the ingestion of antioxidants compared to the placebo conditions (ANOVA time effect, *P* = 0.08). After the sprints performed in hypoxia, the elevation of plasma insulin was marginally greater than in normoxia (F_I_O_2_ × time interaction, *P* = 0.045).

### PDH phosphorylation

**Figure 3:**
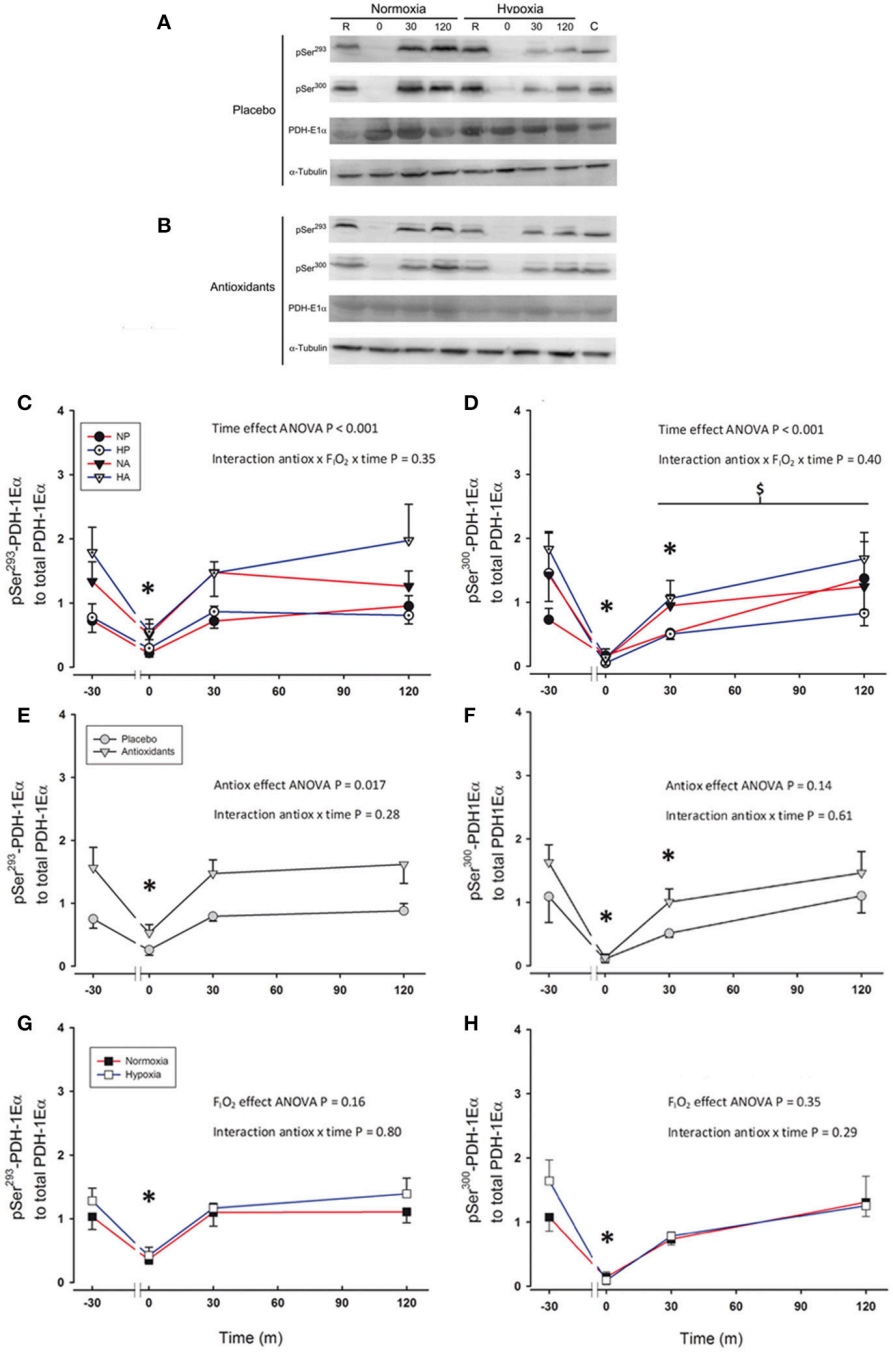
Representative western blots for Ser^293^-PDH-1Eα and Ser^300^-PDH-1Eα phosphorylations and PDH-1Eα total protein expression in response to a single 30 s sprint after placebo **(A)** or antioxidants **(B)** intake. Levels of Ser^293^-PDH-1Eα phosphorylation to PDH-1Eα total protein expression **(C,E,G)**, and Ser^300^-PDH-1Eα phosphorylation to total PDH-1Eα total protein expression **(D,F,H)** before, immediately after, 30 and 120 min following the end of a single 30 s all-out sprint (Wingate test). **(C,D)** Responses to sprints performed in normoxia placebo (black circles), hypoxia placebo (open circles, F_I_O_2_: 0.105), normoxia antioxidants (black triangles) and hypoxia antioxidants (open triangles; F_I_O_2_: 0.105). **(E,F)** Represent the responses observed for two placebo conditions (gray circles) averaged compared to the average of the two antioxidants conditions (gray triangles). **(G,H)** Represent the responses for the average of the normoxic conditions (black squares) compared to the average of the hypoxic conditions (open squares; F_I_O_2_:0.105). ^*^P < 0.05 in comparison to resting (ANOVA, time main effect). ^$^*P* < 0.05 compared to Ser^293^-PDH-1Eα phosphorylation recovery. *n* = 9 for all variables.

Immediately after the sprint Ser^293^-PDH-E1α phosphorylation was reduced to a similar level in all conditions (~66%, *P* = 0.002; Figures 3A–C). However, 30 min after the sprint preceded by the ingestion of antioxidants Ser^293^-PDH-E1α phosphorylation reached pre-exercise values (*P* = 0.86). Ser^293^-PDH-E1α phosphorylation was 93% greater after the ingestion of antioxidants than placebo (ANOVA antioxidants effect *P* = 0.017; Figure 3E). Ser^293^-PDH-E1α was dephosphorylated to similar values immediately after the sprints in normoxia and hypoxia (*P* = 0.68, for the comparison between normoxia vs. hypoxia immediately after the sprints; Figure 3G).

Immediately after the sprints, Ser^300^-PDH-E1α phosphorylation was reduced by 91% (*P* < 0.001), without significant differences between conditions (ANOVA antioxidants × F_I_O_2_ × time *P* = 0.40, Figures 3A,B,D). However, during the recovery period, the re-phosphorylation of Ser^300^ was slower than that of Ser^293^ (ANOVA phosphorylation type × time interaction *P* = 0.012), and 30 min after the end of the sprint Ser^300^-PDH-E1α phosphorylation was still 44% below pre-exercise values (*P* = 0.01). Two hours after the end of the sprint Ser^300^-PDH-E1α phosphorylation reached a value similar to that measured before the sprints (*P* = 0.80). The Ser^300^-PDH-E1α phosphorylation values during the experiment preceded by the ingestion of antioxidants were 50% greater than after the intake of placebo. However, this difference was not statistically significant (*P* = 0.14; Figure 3F). Ser^300^-PDH-E1α phosphorylation changes were similar in the normoxic and hypoxic sprints (ANOVA interaction: *P* = 0.29; Figure 3H).

The area under the curve (AUC) of Ser^293^-PDH-E1α phosphorylation was 87% greater after the ingestion of antioxidants (*P* = 0.01), indicating a greater level of re-phosphorylation during the recovery period after the ingestion of antioxidants. A similar trend was observed for the AUC of the Ser^300^-PDH-E1α phosphorylation (*P* = 0.061).

There was an association between the changes in Ser^293^ and Ser^300^-PDH-E1α phosphorylation observed from pre, to immediately after the sprints in the placebo conditions (*r* = 0.74, *P* < 0.001; *n* = 18), that was lost in the sprints preceded by the ingestion of antioxidants (*r* = 0.35, *P* = 0.15). There was also a negative association between the level of Ser^300^-PDH-E1α phosphorylation at the end of the sprint and the mean and peak power during the sprint (*r* = −0.81, and *r* = −86, respectively, both *P* < 0.01, *n* = 9, each point representing the mean value of the four conditions for each subject). Likewise, a negative association was observed between the immediate post-exercise Ser^293^-PDH-E1α phosphorylation and the corresponding NAD^+^/NADH^+^ ratio (*r* = −0.80, *P* = 0.01, *n* = 9, each point representing the mean value of the four conditions for each subject).

## Discussion

Compared to normoxia, during sprint exercise in severe acute hypoxia the glycolytic rate is increased leading to greater muscle lactate accumulation, acidification and oxidative stress (Morales-Alamo et al., 2012). Here we show that this increased accumulation of lactate is not due to a lower level of PDH activation during the sprint in hypoxia, pointing toward a mitochondrial limitation of the rate of pyruvate decarboxylation to acetyl-CoA and subsequent oxidation of acetyl groups. In addition, by administering a powerful antioxidant cocktail before the sprint we have demonstrated that antioxidants reduce the glycolytic rate and muscle lactate accumulation during the sprints by a mechanism independent of PDH activation, that seems unrelated to F_I_O_2_. We have also demonstrated that after the ingestion of antioxidants, PDH is re-phosphorylated to a higher level, which may facilitate a metabolic shift from carbohydrates to fat oxidation (Sjøberg et al., 2017) during the recovery period, at the expense of delaying the removal of muscle lactate. Our results confirm previous *in vitro* studies showing that the re-phosphorylation of Ser^300^-PDH-E1α is slower than that of Ser^293^-PDH-E1α (Yeaman et al., 1978), indicating a slightly different regulation of these two phosphorylation sites in human skeletal muscle or reflecting intrinsic differences between the two phosphorylations. This is also supported by the negative association observed between the level of Ser^300^-PDH-E1α phosphorylation at the end of the sprint and the mean and peak power during the sprint, which was not observed in the case of Ser^293^-PDH-E1α. The most plausible link between power output and Ser^300^-PDH-E1α dephosphorylation is the increase of intracellular Ca^2+^, which is a fundamental determinant of power output (Bakker et al., 2017).

### PDH dephosphorylation-response to sprint exercise is not modified by antioxidants

The activity of PDH is regulated by phosphorylation/dephosphorylation of three serine residues (Ser^232^, Ser^293^, Ser^300^) of the E1α subunit (Kolobova et al., 2001; Korotchkina and Patel, 2001) (Figure 1). Phosphorylation at any of the three sites leads to inhibition of the complex *in vitro* (Korotchkina and Patel, 1995). These phosphorylations are carried out by pyruvate dehydrogenase kinases, of which four isoforms have been identified (PDK1 to 4), while dephosphorylation is performed by PDH phosphatases, of which two isoforms have been identified (PDP1 and 2) (Teague et al., 1982; Huang et al., 1998). PDK1 is the only isoform reported to phosphorylate all three sites, while PDK2, PDK3, and PDK4 act on Ser^293^ and Ser^300^ *in vitro* (Kolobova et al., 2001; Korotchkina and Patel, 2001). The most abundant PDK in skeletal muscle is the isoform PDK4, followed by the isoform PDK2 and the less abundant is the isoform PDK1. Nevertheless, knockout mice for PDK2 and PDK4 have a constitutively active PDH in skeletal muscles (Rahimi et al., 2014).

**Figure 4:**
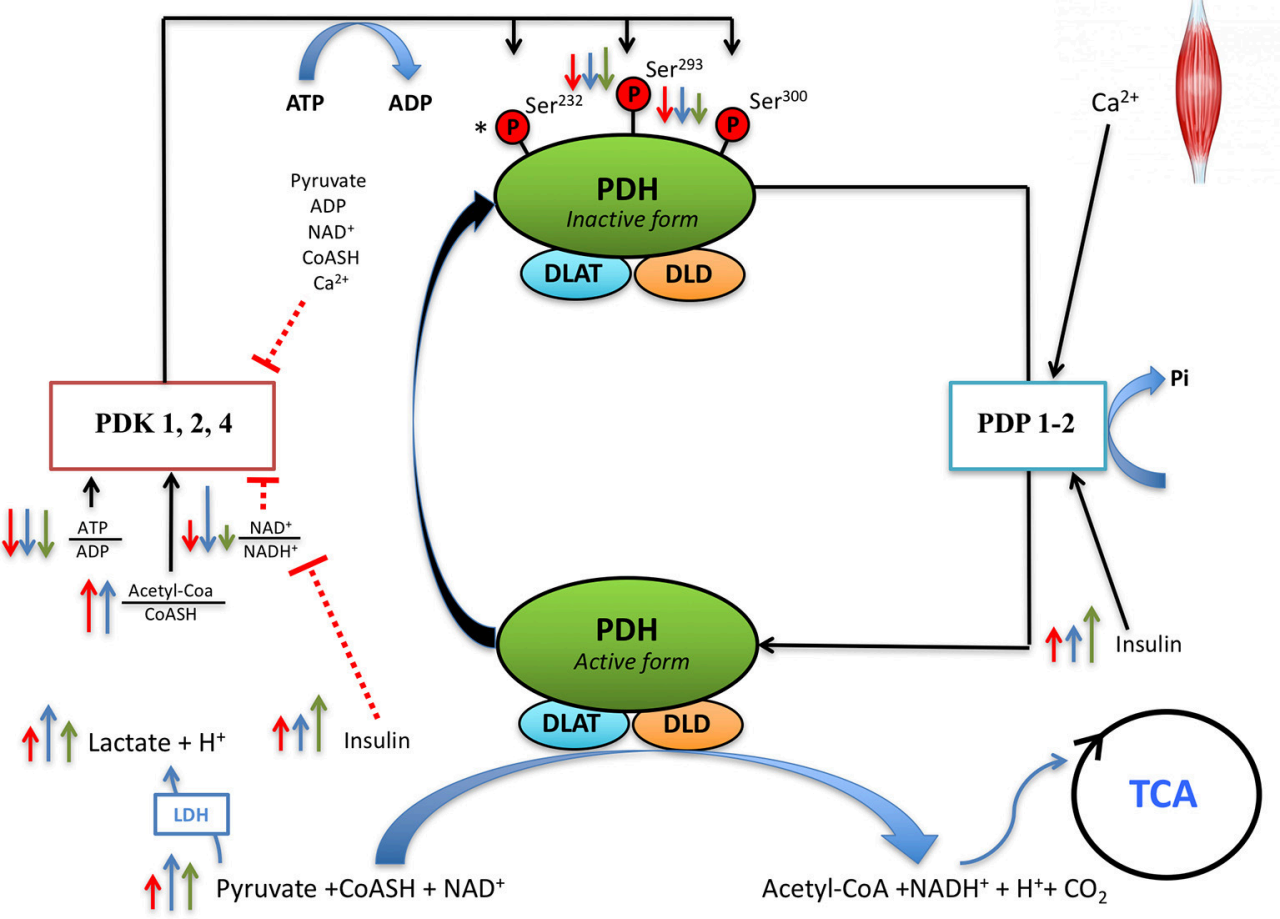
Proposed regulation of PDH activity during sprint exercise and the early post-exercise recovery. During the sprint exercise muscle contractions elicit an immediate increase of sarcoplasmic (Ca^2+^) which activates pyruvate dehydrogenase phosphatase (PDP, mainly PDP1 which is more expressed in skeletal muscle). PDPs dephosphorylate PDH in the serines 293 and 300, regardless of F_I_O_2_. While PDH dephosphorylation during sprint exercise is not modified by antioxidants, antioxidants facilitate Ser^293^PDH-1Eα re-phosphorylation during the recovery period. This indicates that reactive nitrogen and oxygen species (RNOS) contribute maintaining PDH in its active form, or that RNOS slow-down re-phosphorylation during the recovery period. Several regulatory factors of PDKs activity are modified by sprint exercise. After the sprints, the ATP/ADP ratio is decreased to a similar extent in normoxia and hypoxia, regardless of the ingestion of antioxidants. The NAD^+^/NADH^+^ ratio is decreased to a greater extent in hypoxia than normoxia, with this effect being attenuated after the ingestion of antioxidants. Serum insulin increases after the sprint exercise, more when the sprints are performed in hypoxia than normoxia. Normoxia (red arrows), hypoxia (blue arrows), antioxidants (green arrows). The length of the arrow is representative of the magnitude of the change. ^*^Indicates that Ser^232^PDH-1Eα was not measured in the present investigation and, therefore, its regulation by F_I_O_2_ and RNOS during sprint exercise remains unknown.

Previous studies have shown that during sprint exercise in normoxia PDH activity is close to its maximum (Parolin et al., 1999), implying that in normoxia lactate accumulation is due to a limitation in the rate at which pyruvate is oxidized (Howlett et al., 1999). As a novelty, the present investigation shows that PDH is dephosphorylated to a similar extent in normoxia and hypoxia with or without antioxidants (Figure 4). Assuming that Ser^293^ and Ser^300^ PDH phosphorylations can be used to assess PDH activity indirectly (Linn et al., 1969; Pilegaard et al., 2006; Kiilerich et al., 2008, 2010), our data indicate similar levels of activation of PDH at the end of the sprint in all conditions. We have reported that during the sprints in severe acute hypoxia our subjects had higher oxidative stress than in normoxia, as shown by a 50% greater intramuscular protein carbonylation and more marked RNOS-mediated signaling (Morales-Alamo et al., 2012). Moreover, the NAD^+^/NADH^+^ ratio was decreased more after the sprints in hypoxia than normoxia (Morales-Alamo et al., 2012). NADH^+^ is an activator of PDKs (Roche and Hiromasa, 2007), which inhibits PDH through serine phosphorylations (Hucho et al., 1972; Behal et al., 1993). However, PDH dephosphorylation was not greater immediately after the sprint in hypoxia than in normoxia, what is against a potentially greater level of PDK activity during the sprint in hypoxia than in normoxia.

Thus, both in normoxia and hypoxia, the main factor explaining the accumulation of lactate should relate to either an excessive stimulation of glycolysis and/or a limitation of mitochondrial disposal of pyruvate. Given the fact that leg O_2_ delivery and leg O_2_ uptake are reduced during sprint exercise in hypoxia compared to the same exercise in normoxia (Calbet et al., 2015), the greater accumulation of lactate in hypoxia than in normoxia likely reflects a mitochondrial limitation due to insufficient O_2_ supply.

Nevertheless, our data could also indicate that during the sprints in severe acute hypoxia, and perhaps also in normoxia, the stimulation of glycolysis may be excessive in regard with the actual energy demand (Morales-Alamo et al., 2017). This possibility is supported by the fact that during sprint exercise in hypoxia mean power output was not reduced by the ingestion of antioxidants, despite a marked reduction of ATP provision by the glycolysis, that could not be compensated for by increasing VO_2_, since during sprint exercise in hypoxia VO_2_ was the same in the placebo and the antioxidants conditions. This finding contrasts with the reported reduction of muscle strength and muscle VO_2_ after the intravenous administration of vitamin C and tempol in rats (Herspring et al., 2008). Moreover, the fact that mean power output was not reduced after the administration of antioxidants in acute hypoxia is intriguing because the glycolysis provides 50% or more of the overall energy expenditure during the Wingate test in normoxia (Bogdanis et al., 1996; Parolin et al., 1999) and even more during sprint exercise in hypoxia (Morales-Alamo et al., 2012). Therefore, our antioxidant cocktail should have contributed to reduce the energy demand by, for example, improving the P/O ratio and/or by lowering the energy cost of muscle contraction.

Mitochondria produces RNOS mainly at complexes I and III of the electron transport chain (Barja, 1999; Muller et al., 2004). Ascorbate prevents cytochrome *c* release from the mitochondrial inner membrane and stabilizes the mitochondrial membrane potential in response to hypoxia-reoxygenation (Dhar-Mascareño et al., 2005) and could improve the phosphorylation potential by increasing the proton motive force across the mitochondrial inner membrane (Brand and Nicholls, 2011). The presence of α-lipoic acid in our antioxidant cocktail could have amplified this effect (Cimolai et al., 2014). Moreover, in agreement with an improved mitochondrial efficiency, experiments in humans, have reported enhanced mitochondrial efficiency during exercise in moderate hypoxia (F_I_O_2_: 0.13) after nitrate supplementation (Vanhatalo et al., 2014). Antioxidants, like nitrates, increase NO bioavailability (Nyberg et al., 2012; Larsen et al., 2014), which can reduce proton back-leakage across the inner mitochondrial membrane increasing the P/O ratio (Clerc et al., 2007).

Antioxidants may also reduce the energy demand. One of the main contributors to energy expenditure during muscle contraction is the sarcoendoplasmic reticulum calcium pumps (SERCAs), which may account for ~50% of the overall energy expenditure at rest (Norris et al., 2010) and ~30–40% during muscle contractions (Barclay et al., 2007). SERCAs-energy expenditure can be lowered by reducing the amount of calcium efflux through the calcium channels [the ryanodine receptor and the inositol 1, 4, 5-trisphosphate receptors (IP3Rs)] and/or by decreasing Ca^2+^ leakage from the sarcoendoplasmic reticulum (Chernorudskiy and Zito, 2017). Both processes are increased by RNOS and attenuated by antioxidants (Xu et al., 1998; Kang et al., 2008; Mazurek et al., 2014; Oda et al., 2015). Nevertheless, excessive oxidative stress may also reduce SERCAs activity (Chernorudskiy and Zito, 2017). Calcium transients were likely reduced during the sprints with antioxidants since CaMKII, a kinase that auto-phosphorylates depending on the magnitude of Ca^2+^ transients was less phosphorylated after the sprints preceded by the ingestion of antioxidants in our subjects (Morales-Alamo et al., 2013, 2017).

### PDH re-phosphorylation during the recovery period is increased by antioxidants

The recovery of the resting PDH phosphorylation levels requires the activation of PDKs by acetyl-CoA, ${\text{NADH}}_{,}^{+}$ or ATP, which re-phosphorylate PDH favoring the oxidation of acetyl groups (Roche and Hiromasa, 2007; Rahimi et al., 2014). Here we show that antioxidants facilitate PDH re-phosphorylation during the recovery period, with faster re-phosphorylation of Ser^293^ than Ser^300^ (Figure 4), as previously shown with experiments *in vitro* (Yeaman et al., 1978). Cell-culture experiments have shown that antioxidants stimulate PDKs (Churchill et al., 2005). Although not measured here, PDK activity during the recovery period might have been stimulated by the lower NAD^+^/NADH^+^ ratio after the ingestion of antioxidants (Figure 4), as previously reported (Morales-Alamo et al., 2013, 2017). Cell-culture experiments have also shown that pyruvate dehydrogenase phosphatase 1 (PDP1) activity is stimulated by lipoic acid (Shan et al., 2014). Nevertheless, an enhanced α-lipoic acid-stimulated PDP1 activity after the ingestion of antioxidants is unlikely inasmuch PDH re-phosphorylation was facilitated during the recovery period after the ingestion of antioxidants. Although PDPs are stimulated by insulin (Thomas and Denton, 1986; Consitt et al., 2016), the insulin response to sprint exercise was not reduced after ingestion of antioxidants in the present study. Thus, the greater PDH re-phosphorylation during the recovery period after antioxidants cannot be attributed to an effect mediated by differences in the plasma insulin concentrations, since our results point toward higher insulin concentrations after the administration of antioxidants, which via PDP activation could delay, but not accelerate, PDH re-phosphorylation.

Another mechanism that could explain a faster re-phosphorylation of PDH during the recovery period after the ingestion of antioxidants is a reduction of the exercise-induced increase in insulin sensitivity by antioxidants (Trewin et al., 2015). Indeed, it has been reported that the increase of insulin sensitivity observed acutely after exercise is augmented in mice lacking the antioxidant enzyme Gpx1, an effect that was abolished by administration of the antioxidant N-acetylcysteine (Loh et al., 2009). This concurs with the lower post-exercise insulin sensitivity observed in humans who received N-acetylcysteine during exercise (Trewin et al., 2015).

As expected from a greater re-phosphorylation of PDH (i.e., inhibition of PDH) following the ingestion of antioxidants, muscle lactate concentration 30 min after the end of the sprints was almost twice as high after the ingestion of antioxidants. This likely reflects a lower rate of lactate oxidation during the recovery after the ingestion of antioxidants. Lactate oxidation must be preceded by the conversion of lactate into pyruvate and subsequently decarboxylated to acetyl-CoA by the PDH, which after the ingestion of antioxidants is less active (Gladden, 2006). Consequently, to maintain the flow of acetyl groups entering the Krebs cycle and the aerobic ATP resynthesis rate during the first 30–60 min after a sprint exercise preceded by the ingestion of antioxidants, fat oxidation should be increased.

### Why was performance not improved by antioxidants, despite the attenuation of muscle lactate accumulation and acidification?

Our findings are at odds with the current paradigm of muscle fatigue (Fitts, 1994; Bangsbo and Juel, 2006), but concur with alternative proposals based on human and animal experiments (Nielsen et al., 2001; Lamb and Stephenson, 2006; Morales-Alamo et al., 2015; Torres-Peralta et al., 2016a,b). Muscle fatigue during high-intensity exercise has been traditionally linked to the activation of glycolysis, the accumulation of lactate and the effects caused by the drop in muscle pH (Fitts, 1994). However, *in vitro* and animal experiments have shown that both intracellular acidification and lactate accumulation contribute to maintain muscle excitability attenuating fatigue (Westerblad et al., 1991; Allen et al., 1995; Pedersen et al., 2004, 2005). Specifically, lactate can inhibit ClC-1 Cl^−^ channels and increase the excitability and contractile function of depolarized rat muscles (de Paoli et al., 2010). More recently, the ergogenic effect of lactate accumulation has been confirmed in humans, which recovered partly from fatigue during 1 min of completed bilateral leg occlusion applied at the end of an incremental exercise to exhaustion, despite a 19–22% increase of muscle lactate during the ischemic recovery (Morales-Alamo et al., 2015). In the current investigation, peak power output was not affected by either hypoxia or antioxidants, while mean power output was only reduced by 5.3% in hypoxia (all conditions combined), despite a much greater accumulation of muscle lactate. The fact that muscle lactate could accumulate to higher levels with a minor impact on performance agrees with the idea that lactate and acidification is not as detrimental for muscle contraction as classically thought. However, the potential protective effect on muscle excitability by greater lactate accumulation during exercise in severe acute hypoxia was lost when the sprints were preceded by the intake of antioxidants, which reduced the glycolytic rate to values similar to those observed during the sprints performed in normoxia. This had a minor impact on performance and we think that our findings are compatible with mechanisms of task failure localized outside the active skeletal muscles (Calbet et al., 2015; Morales-Alamo et al., 2015; Torres-Peralta et al., 2016a,b).

In summary, this investigation shows that lactate accumulation during sprint exercise in severe acute hypoxia is not caused by reduced activation of the PDH, which dephosphorylates to similar levels in normoxia and hypoxia. The ingestion of antioxidants before sprint exercise reduces the glycolytic rate in normoxia and hypoxia without a negative impact on performance, implying that either the activation of glycolysis in hypoxia is excessive and/or the energy cost of muscle contraction is reduced by antioxidants. We have also shown that Ser^293^ re-phosphorylates a faster rate during the recovery period than Ser^300^-PDH-E1α, suggesting slightly different regulatory mechanisms that remain to be identified. Finally, the ingestion of antioxidants is associated with increased PDH re-phosphorylation and slower elimination of muscle lactate during the recovery period.

## Author contributions

JAC, DM-A, BG, and CD: Conception and design of the experiments; JAC, DM-A, BG, AS, and CD: Pre-testing, experimental preparation, and data collection; All co-authors: data analysis. The first draft of the manuscript was written by DM-A and JAC. All co-authors edited and proofread the manuscript and approved the final version.

### Conflict of interest statement

The authors declare that the research was conducted in the absence of any commercial or financial relationships that could be construed as a potential conflict of interest.

## References

[B1] Allen D. G. Westerblad H. Lännergren J. (1995). The role of intracellular acidosis in muscle fatigue. Adv. Exp. Med. Biol. 384, 57–68. 10.1007/978-1-4899-1016-5_5 8585477

[B2] Bailey D. M. Lawrenson L. McEneny J. Young I. S. James P. E. Jackson S. K.. (2007). Electron paramagnetic spectroscopic evidence of exercise-induced free radical accumulation in human skeletal muscle. Free Radic. Res. 41, 182–190. 10.1080/10715760601028867 17364944

[B3] Bakker A. J. Cully T. R. Wingate C. D. Barclay C. J. Launikonis B. S. (2017). Doublet stimulation increases Ca^2+^ binding to troponin C to ensure rapid force development in skeletal muscle. J. Gen. Physiol. 149, 323–334. 10.1085/jgp.201611727 28209802 PMC5339514

[B4] Bangsbo J. Juel C. (2006). Counterpoint: lactic acid accumulation is a disadvantage during muscle activity. J. Appl. Physiol. 100, 1412–1413. discussion: 1413–1414. 16646130 10.1152/japplphysiol.00023.2006

[B5] Barclay C. J. Woledge R. C. Curtin N. A. (2007). Energy turnover for Ca^2+^ cycling in skeletal muscle. J. Muscle Res. Cell Motil. 28, 259–274. 10.1007/s10974-007-9116-7 17882515

[B6] Barja G. (1999). Mitochondrial oxygen radical generation and leak: sites of production in states 4 and 3, organ specificity, and relation to aging and longevity. J. Bioenerg. Biomembr. 31, 347–366. 10.1023/A:1005427919188 10665525

[B7] Bass J. J. Wilkinson D. J. Rankin D. Phillips B. E. Szewczyk N. J. Smith K.. (2017). An overview of technical considerations for Western blotting applications to physiological research. Scand. J. Med. Sci. Sports 27, 4–25. 10.1111/sms.12702 27263489 PMC5138151

[B8] Behal R. H. Buxton D. B. Robertson J. G. Olson M. S. (1993). Regulation of the pyruvate dehydrogenase multienzyme complex. Annu. Rev. Nutr. 13, 497–520. 10.1146/annurev.nu.13.070193.002433 8369156

[B9] Bogdanis G. C. Nevill M. E. Boobis L. H. Lakomy H. K. (1996). Contribution of phosphocreatine and aerobic metabolism to energy supply during repeated sprint exercise. J. Appl. Physiol. 80, 876–884. 10.1152/jappl.1996.80.3.876 8964751

[B10] Brand M. D. Nicholls D. G. (2011). Assessing mitochondrial dysfunction in cells. Biochem. J. 435, 297–312. 10.1042/BJ20110162 21726199 PMC3076726

[B11] Brennan-Minnella A. M. Won S. J. Swanson R. A. (2015). NADPH oxidase-2: linking glucose, acidosis, and excitotoxicity in stroke. Antioxid. Redox Signal. 22, 161–174. 10.1089/ars.2013.5767 24628477 PMC4281853

[B12] Calbet J. A. Chavarren J. Dorado C. (1997). Fractional use of anaerobic capacity during a 30- and a 45-s Wingate test. Eur. J. Appl. Physiol. 76, 308–313. 10.1007/s004210050253 9349644

[B13] Calbet J. A. De Paz J. A. Garatachea N. Cabeza De Vaca S. Chavarren J. (2003). Anaerobic energy provision does not limit Wingate exercise performance in endurance-trained cyclists. J. Appl. Physiol. 94, 668–676. 10.1152/japplphysiol.00128.2002 12391104

[B14] Calbet J. A. Losa-Reyna J. Torres-Peralta R. Rasmussen P. Ponce-Gonzalez J. G. Sheel A. W.. (2015). Limitations to oxygen transport and utilization during sprint exercise in humans: evidence for a functional reserve in muscle O_2_ diffusing capacity. J. Physiol. 593, 4649–4664. 10.1113/JP270408 26258623 PMC4606532

[B15] Calbet J. A. Moysi J. S. Dorado C. Rodríguez L. P. (1998). Bone mineral content and density in professional tennis players. Calcif. Tissue Int. 62, 491–496. 10.1007/s002239900467 9576975

[B16] Chasiotis D. Sahlin K. Hultman E. (1982). Regulation of glycogenolysis in human muscle at rest and during exercise. J. Appl. Physiol. Respir. Environ. Exerc. Physiol. 53, 708–715. 10.1152/jappl.1982.53.3.708 6813302

[B17] Cheetham M. E. Boobis L. H. Brooks S. Williams C. (1986). Human muscle metabolism during sprint running. J. Appl. Physiol. 61, 54–60. 10.1152/jappl.1986.61.1.54 3733627

[B18] Chernorudskiy A. L. Zito E. (2017). Regulation of calcium homeostasis by ER redox: a close-up of the ER/Mitochondria connection. J. Mol. Biol. 429, 620–632. 10.1016/j.jmb.2017.01.017 28137421

[B19] Churchill E. N. Murriel C. L. Chen C. H. Mochly-Rosen D. Szweda L. I. (2005). Reperfusion-induced translocation of deltaPKC to cardiac mitochondria prevents pyruvate dehydrogenase reactivation. Circ. Res. 97, 78–85. 10.1161/01.RES.0000173896.32522.6e 15961716

[B20] Cimolai M. C. Vanasco V. Marchini T. Magnani N. D. Evelson P. Alvarez S. (2014). α-Lipoic acid protects kidney from oxidative stress and mitochondrial dysfunction associated to inflammatory conditions. Food Funct. 5, 3143–3150. 10.1039/C4FO00489B 25272049

[B21] Clerc P. Rigoulet M. Leverve X. Fontaine E. (2007). Nitric oxide increases oxidative phosphorylation efficiency. J. Bioenerg. Biomembr. 39, 158–166. 10.1007/s10863-007-9074-1 17447126

[B22] Consitt L. A. Saxena G. Saneda A. Houmard J. A. (2016). Age-related impairments in skeletal muscle PDH phosphorylation and plasma lactate are indicative of metabolic inflexibility and the effects of exercise training. Am. J. Physiol. Endocrinol. Metab. 311, E145–E156. 10.1152/ajpendo.00452.2015 27221120 PMC4967149

[B23] Cuevas M. J. Almar M. García-Glez J. C. García-López D. De Paz J. A. Alvear-Ordenes I.. (2005). Changes in oxidative stress markers and NF-κB activation induced by sprint exercise. Free Radic. Res. 39, 431–439. 10.1080/10715760500072149 16028368

[B24] de Paoli F. V. Ørtenblad N. Pedersen T. H. Jørgensen R. Nielsen O. B. (2010). Lactate *per se* improves the excitability of depolarized rat skeletal muscle by reducing the Cl-conductance. J. Physiol. 588, 4785–4794. 10.1113/jphysiol.2010.196568 20876199 PMC3010146

[B25] Dhar-Mascareño M. Cárcamo J. M. Golde D. W. (2005). Hypoxia-reoxygenation-induced mitochondrial damage and apoptosis in human endothelial cells are inhibited by vitamin C. Free Radic. Biol. Med. 38, 1311–1322. 10.1016/j.freeradbiomed.2005.01.017 15855049

[B26] Dulhunty A. F. Wei-LaPierre L. Casarotto M. G. Beard N. A. (2017). Core skeletal muscle ryanodine receptor calcium release complex. Clin. Exp. Pharmacol. Physiol. 44, 3–12. 10.1111/1440-1681.12676 27696487

[B27] Fitts R. H. (1994). Cellular mechanisms of muscle fatigue. Physiol. Rev. 74, 49–94. 10.1152/physrev.1994.74.1.49 8295935

[B28] Gladden L. B. (2006). Mammalian skeletal muscle can convert lactate to glycogen. J. Appl. Physiol. 100:2109. 10.1152/japplphysiol.00163.2006 16714420

[B29] Gorostiaga E. M. Navarro-Amézqueta I. Cusso R. Hellsten Y. Calbet J. A. Guerrero M.. (2010). Anaerobic energy expenditure and mechanical efficiency during exhaustive leg press exercise. PLoS ONE 5:e13486. 10.1371/journal.pone.0013486 20976067 PMC2957441

[B30] Guerra B. Gomez-Cabrera M. C. Ponce-Gonzalez J. G. Martinez-Bello V. E. Guadalupe-Grau A. Santana A. (2011). Repeated muscle biopsies through a single skin incision do not elicit muscle signaling, but IL-6 mRNA and STAT3 phosphorylation increase in injured muscle. J. Appl. Physiol. 110, 1708–1715. 10.1152/japplphysiol.00091.2011 21436461

[B31] Guerra B. Guadalupe-Grau A. Fuentes T. Ponce-González J. G. Morales-Alamo D. Olmedillas H.. (2010). SIRT1, AMP-activated protein kinase phosphorylation and downstream kinases in response to a single bout of sprint exercise: influence of glucose ingestion. Eur. J. Appl. Physiol. 109, 731–743. 10.1007/s00421-010-1413-y 20217115

[B32] Guerra B. Santana A. Fuentes T. Delgado-Guerra S. Cabrera-Socorro A. Dorado C.. (2007). Leptin receptors in human skeletal muscle. J. Appl. Physiol. 102, 1786–1792. 10.1152/japplphysiol.01313.2006 17234799

[B33] Harris R. C. Edwards R. H. Hultman E. Nordesjö L. O. Nylind B. Sahlin K. (1976). The time course of phosphorylcreatine resynthesis during recovery of the quadriceps muscle in man. Pflugers Arch. 367, 137–142. 10.1007/BF00585149 1034909

[B34] Herspring K. F. Ferreira L. F. Copp S. W. Snyder B. S. Poole D. C. Musch T. I. (2008). Effects of antioxidants on contracting spinotrapezius muscle microvascular oxygenation and blood flow in aged rats. J. Appl. Physiol. 105, 1889–1896. 10.1152/japplphysiol.90642.2008 18845782

[B35] Howlett R. A. Heigenhauser G. J. Spriet L. L. (1999). Skeletal muscle metabolism during high-intensity sprint exercise is unaffected by dichloroacetate or acetate infusion. J. Appl. Physiol. 87, 1747–1751. 10.1152/jappl.1999.87.5.1747 10562618

[B36] Huang B. Gudi R. Wu P. Harris R. A. Hamilton J. Popov K. M. (1998). Isoenzymes of pyruvate dehydrogenase phosphatase. DNA-derived amino acid sequences, expression, and regulation. J. Biol. Chem. 273, 17680–17688. 10.1074/jbc.273.28.17680 9651365

[B37] Hucho F. Randall D. D. Roche T. E. Burgett M. W. Pelley J. W. Reed L. J. (1972). α-Keto acid dehydrogenase complexes. XVII. Kinetic and regulatory properties of pyruvate dehydrogenase kinase and pyruvate dehydrogenase phosphatase from bovine kidney and heart. Arch. Biochem. Biophys. 151, 328–340. 10.1016/0003-9861(72)90504-8 4339797

[B38] Jackson M. J. (2015). Redox regulation of muscle adaptations to contractile activity and aging. J. Appl. Physiol. 119, 163–171. 10.1152/japplphysiol.00760.2014 25792715 PMC4526708

[B39] Jackson M. J. Pye D. Palomero J. (2007). The production of reactive oxygen and nitrogen species by skeletal muscle. J. Appl. Physiol. 102, 1664–1670. 10.1152/japplphysiol.01102.2006 17082364

[B40] Jones N. L. McCartney N. Graham T. Spriet L. L. Kowalchuk J. M. Heigenhauser G. J.. (1985). Muscle performance and metabolism in maximal isokinetic cycling at slow and fast speeds. J. Appl. Physiol. 59, 132–136. 10.1152/jappl.1985.59.1.132 4030556

[B41] Kang S. Kang J. Kwon H. Frueh D. Yoo S. H. Wagner G.. (2008). Effects of redox potential and Ca^2+^ on the inositol 1,4,5-trisphosphate receptor L3-1 loop region: implications for receptor regulation. J. Biol. Chem. 283, 25567–25575. 10.1074/jbc.M803321200 18635540

[B42] Katz A. Sahlin K. Henriksson J. (1986). Muscle ammonia metabolism during isometric contraction in humans. Am. J. Physiol. 250, C834–C840. 10.1152/ajpcell.1986.250.6.C834 2872818

[B43] Kiilerich K. Birk J. B. Damsgaard R. Wojtaszewski J. F. Pilegaard H. (2008). Regulation of PDH in human arm and leg muscles at rest and during intense exercise. Am. J. Physiol. Endocrinol. Metab. 294, E36–E42. 10.1152/ajpendo.00352.2007 17957032

[B44] Kiilerich K. Gudmundsson M. Birk J. B. Lundby C. Taudorf S. Plomgaard P. (2010). Low muscle glycogen and elevated plasma free fatty acid modify but do not prevent exercise-induced PDH activation in human skeletal muscle. Diabetes 59, 26–32. 10.2337/db09-1032 19833896 PMC2797931

[B45] Kolobova E. Tuganova A. Boulatnikov I. Popov K. M. (2001). Regulation of pyruvate dehydrogenase activity through phosphorylation at multiple sites. Biochem. J. 358, 69–77. 10.1042/bj3580069 11485553 PMC1222033

[B46] Korotchkina L. G. Patel M. S. (1995). Mutagenesis studies of the phosphorylation sites of recombinant human pyruvate dehydrogenase. Site-specific regulation. J. Biol. Chem. 270, 14297–14304. 10.1074/jbc.270.24.14297 7782287

[B47] Korotchkina L. G. Patel M. S. (2001). Site specificity of four pyruvate dehydrogenase kinase isoenzymes toward the three phosphorylation sites of human pyruvate dehydrogenase. J. Biol. Chem. 276, 37223–37229. 10.1074/jbc.M103069200 11486000

[B48] Lamb G. D. Stephenson D. G. (2006). Point: lactic acid accumulation is an advantage during muscle activity. J. Appl. Physiol. 100, 1410–1412. discussion: 1414. 10.1152/japplphysiol.00023.2006 16540714

[B49] Larsen F. J. Schiffer T. A. Ekblom B. Mattsson M. P. Checa A. Wheelock C. E.. (2014). Dietary nitrate reduces resting metabolic rate: a randomized, crossover study in humans. Am. J. Clin. Nutr. 99, 843–850. 10.3945/ajcn.113.079491 24500154

[B50] Linn T. C. Pettit F. H. Reed L. J. (1969). Alpha-keto acid dehydrogenase complexes. X. Regulation of the activity of the pyruvate dehydrogenase complex from beef kidney mitochondria by phosphorylation and dephosphorylation. Proc. Natl. Acad. Sci. U.S.A. 62, 234–241. 10.1073/pnas.62.1.234 4306045 PMC285978

[B51] Loh K. Deng H. Fukushima A. Cai X. Boivin B. Galic S.. (2009). Reactive oxygen species enhance insulin sensitivity. Cell Metab. 10, 260–272. 10.1016/j.cmet.2009.08.009 19808019 PMC2892288

[B52] Lowry O. H. Passonneau J. V. (1972). A Flexible System of Enzymatic Analysis. New York, NY: Academic Press.

[B53] Mazurek S. R. Bovo E. Zima A. V. (2014). Regulation of sarcoplasmic reticulum Ca(2+) release by cytosolic glutathione in rabbit ventricular myocytes. Free Radic. Biol. Med. 68, 159–167. 10.1016/j.freeradbiomed.2013.12.003 24334252

[B54] McLellan T. M. Kavanagh M. F. Jacobs I. (1990). The effect of hypoxia on performance during 30 s or 45 s of supramaximal exercise. Eur. J. Appl. Physiol. Occup. Physiol. 60, 155–161. 10.1007/BF00846037 2335174

[B55] Morales-Alamo D. Calbet J. A. (2014). Free radicals and sprint exercise in humans. Free Radic. Res. 48, 30–42. 10.3109/10715762.2013.825043 23879691

[B56] Morales-Alamo D. Guerra B. Ponce-González J. G. Guadalupe-Grau A. Santana A. Martin-Rincon M.. (2017). Skeletal muscle signaling, metabolism, and performance during sprint exercise in severe acute hypoxia after the ingestion of antioxidants. J. Appl. Physiol. 123, 1235–1245. 10.1152/japplphysiol.00384.2017 28819003

[B57] Morales-Alamo D. Losa-Reyna J. Torres-Peralta R. Martin-Rincon M. Perez-Valera M. Curtelin D.. (2015). What limits performance during whole-body incremental exercise to exhaustion in humans? J. Physiol. 593, 4631–4648. 10.1113/JP270487 26250346 PMC4606539

[B58] Morales-Alamo D. Ponce-González J. G. Guadalupe-Grau A. Rodríguez-García L. Santana A. Cusso M. R.. (2012). Increased oxidative stress and anaerobic energy release, but blunted Thr172-AMPKα phosphorylation, in response to sprint exercise in severe acute hypoxia in humans. J. Appl. Physiol. 113, 917–928. 10.1152/japplphysiol.00415.2012 22858621

[B59] Morales-Alamo D. Ponce-González J. G. Guadalupe-Grau A. Rodríguez-García L. Santana A. Cusso R.. (2013). Critical role for free radicals on sprint exercise-induced CaMKII and AMPKα phosphorylation in human skeletal muscle. J. Appl. Physiol. 114, 566–577. 10.1152/japplphysiol.01246.2012 23288553

[B60] Morgan D. Cherny V. V. Murphy R. Katz B. Z. DeCoursey T. E. (2005). The pH dependence of NADPH oxidase in human eosinophils. J. Physiol. 569, 419–431. 10.1113/jphysiol.2005.094748 16195320 PMC1464255

[B61] Muller F. L. Liu Y. Van Remmen H. (2004). Complex III releases superoxide to both sides of the inner mitochondrial membrane. J. Biol. Chem. 279, 49064–49073. 10.1074/jbc.M407715200 15317809

[B62] Nielsen O. B. de Paoli F. Overgaard K. (2001). Protective effects of lactic acid on force production in rat skeletal muscle. J. Physiol. 536, 161–166. 10.1111/j.1469-7793.2001.t01-1-00161.x 11579166 PMC2278832

[B63] Norris S. M. Bombardier E. Smith I. C. Vigna C. Tupling A. R. (2010). ATP consumption by sarcoplasmic reticulum Ca^2+^ pumps accounts for 50% of resting metabolic rate in mouse fast and slow twitch skeletal muscle. Am. J. Physiol. Cell Physiol. 298, C521–C529. 10.1152/ajpcell.00479.2009 20018953

[B64] Nyberg M. Blackwell J. R. Damsgaard R. Jones A. M. Hellsten Y. Mortensen S. P. (2012). Lifelong physical activity prevents an age-related reduction in arterial and skeletal muscle nitric oxide bioavailability in humans. J. Physiol. 590, 5361–5370. 10.1113/jphysiol.2012.239053 22890714 PMC3515824

[B65] Oda T. Yang Y. Uchinoumi H. Thomas D. D. Chen-Izu Y. Kato T.. (2015). Oxidation of ryanodine receptor (RyR) and calmodulin enhance Ca release and pathologically alter, RyR structure and calmodulin affinity. J. Mol. Cell. Cardiol. 85, 240–248. 10.1016/j.yjmcc.2015.06.009 26092277 PMC4530019

[B66] Parolin M. L. Chesley A. Matsos M. P. Spriet L. L. Jones N. L. Heigenhauser G. J. (1999). Regulation of skeletal muscle glycogen phosphorylase and PDH during maximal intermittent exercise. Am. J. Physiol. 277, E890–E900. 10.1152/ajpendo.1999.277.5.E890 10567017

[B67] Parolin M. L. Spriet L. L. Hultman E. Hollidge-Horvat M. G. Jones N. L. Heigenhauser G. J. (2000). Regulation of glycogen phosphorylase and PDH during exercise in human skeletal muscle during hypoxia. Am. J. Physiol. Endocrinol. Metab. 278, E522–E534. 10.1152/ajpendo.2000.278.3.E522 10710508

[B68] Parra J. Cadefau J. A. Rodas G. Amigó N. Cussó R. (2000). The distribution of rest periods affects performance and adaptations of energy metabolism induced by high-intensity training in human muscle. Acta Physiol. Scand. 169, 157–165. 10.1046/j.1365-201x.2000.00730.x 10848646

[B69] Pedersen T. H. de Paoli F. Nielsen O. B. (2005). Increased excitability of acidified skeletal muscle: role of chloride conductance. J. Gen. Physiol. 125, 237–246. 10.1085/jgp.200409173 15684096 PMC2217490

[B70] Pedersen T. H. Nielsen O. B. Lamb G. D. Stephenson D. G. (2004). Intracellular acidosis enhances the excitability of working muscle. Science 305, 1144–1147. 10.1126/science.1101141 15326352

[B71] Pilegaard H. Birk J. B. Sacchetti M. Mourtzakis M. Hardie D. G. Stewart G.. (2006). PDH-E1α dephosphorylation and activation in human skeletal muscle during exercise: effect of intralipid infusion. Diabetes 55, 3020–3027. 10.2337/db06-0152 17065338

[B72] Putman C. T. Jones N. L. Lands L. C. Bragg T. M. Hollidge-Horvat M. G. Heigenhauser G. J. (1995). Skeletal muscle pyruvate dehydrogenase activity during maximal exercise in humans. Am. J. Physiol. 269, E458–E468. 10.1152/ajpendo.1995.269.3.E458 7573423

[B73] Radak Z. Zhao Z. Koltai E. Ohno H. Atalay M. (2013). Oxygen consumption and usage during physical exercise: the balance between oxidative stress and ROS-dependent adaptive signaling. Antioxid. Redox Signal. 18, 1208–1246. 10.1089/ars.2011.4498 22978553 PMC3579386

[B74] Rahimi Y. Camporez J. P. Petersen M. C. Pesta D. Perry R. J. Jurczak M. J.. (2014). Genetic activation of pyruvate dehydrogenase alters oxidative substrate selection to induce skeletal muscle insulin resistance. Proc. Natl. Acad. Sci. U.S.A. 111, 16508–16513. 10.1073/pnas.1419104111 25368185 PMC4246337

[B75] Richardson R. S. Donato A. J. Uberoi A. Wray D. W. Lawrenson L. Nishiyama S.. (2007). Exercise-induced brachial artery vasodilation: role of free radicals. Am. J. Physiol. Heart Circ. Physiol. 292, H1516–H1522. 10.1152/ajpheart.01045.2006 17114239

[B76] Roche T. E. Hiromasa Y. (2007). Pyruvate dehydrogenase kinase regulatory mechanisms and inhibition in treating diabetes, heart ischemia, and cancer. Cell. Mol. Life Sci. 64, 830–849. 10.1007/s00018-007-6380-z 17310282 PMC11136253

[B77] Shan C. Kang H. B. Elf S. Xie J. Gu T. L. Aguiar M.. (2014). Tyr-94 phosphorylation inhibits pyruvate dehydrogenase phosphatase 1 and promotes tumor growth. J. Biol. Chem. 289, 21413–21422. 10.1074/jbc.M114.581124 24962578 PMC4118105

[B78] Simchowitz L. (1985). Intracellular pH modulates the generation of superoxide radicals by human neutrophils. J. Clin. Invest. 76, 1079–1089. 10.1172/JCI112061 2995444 PMC423992

[B79] Sjøberg K. A. Frøsig C. Kjøbsted R. Sylow L. Kleinert M. Betik A. C.. (2017). Exercise increases human skeletal muscle insulin sensitivity via coordinated increases in microvascular perfusion and molecular signaling. Diabetes 66, 1501–1510. 10.2337/db16-1327 28292969

[B80] Smith P. K. Krohn R. I. Hermanson G. T. Mallia A. K. Gartner F. H. Provenzano M. D.. (1985). Measurement of protein using bicinchoninic acid. Anal. Biochem. 150, 76–85. 10.1016/0003-2697(85)90442-7 3843705

[B81] Tabatabaie T. Potts J. D. Floyd R. A. (1996). Reactive oxygen species-mediated inactivation of pyruvate dehydrogenase. Arch. Biochem. Biophys. 336, 290–296. 10.1006/abbi.1996.0560 8954577

[B82] Teague W. M. Pettit F. H. Wu T. L. Silberman S. R. Reed L. J. (1982). Purification and properties of pyruvate dehydrogenase phosphatase from bovine heart and kidney. Biochemistry 21, 5585–5592. 10.1021/bi00265a031 6293549

[B83] Thomas A. P. Denton R. M. (1986). Use of toluene-permeabilized mitochondria to study the regulation of adipose tissue pyruvate dehydrogenase *in situ*. Further evidence that insulin acts through stimulation of pyruvate dehydrogenase phosphate phosphatase. Biochem. J. 238, 93–101. 10.1042/bj2380093 3026348 PMC1147101

[B84] Torres-Peralta R. Losa-Reyna J. Morales-Alamo D. González-Izal M. Pérez-Suárez I. Ponce-González J. G.. (2016a). Increased P_I_O_2_ at exhaustion in hypoxia enhances muscle activation and swiftly relieves fatigue: a placebo or a P_I_O_2_ dependent effect? Front. Physiol. 7:333. 10.3389/fphys.2016.00333 27582710 PMC4987359

[B85] Torres-Peralta R. Morales-Alamo D. Gonzalez-Izal M. Losa-Reyna J. Perez-Suarez I. Izquierdo M. (2016b). Task failure during exercise to exhaustion in normoxia and hypoxia is due to reduced muscle activation caused by central mechanisms while muscle metaboreflex does not limit performance. Front. Physiol. 6:414 10.3389/fphys.2015.00414 26793117 PMC4707284

[B86] Trewin A. J. Lundell L. S. Perry B. D. Patil K. V. Chibalin A. V. Levinger I.. (2015). Effect of N-acetylcysteine infusion on exercise-induced modulation of insulin sensitivity and signaling pathways in human skeletal muscle. Am. J. Physiol. Endocrinol. Metab. 309, E388–E397. 10.1152/ajpendo.00605.2014 26105008

[B87] Vanhatalo A. Jones A. M. Blackwell J. R. Winyard P. G. Fulford J. (2014). Dietary nitrate accelerates postexercise muscle metabolic recovery and O_2_ delivery in hypoxia. J. Appl. Physiol. 117, 1460–1470. 10.1152/japplphysiol.00096.2014 25301896 PMC4269683

[B88] Westerblad H. Lee J. A. Lännergren J. Allen D. G. (1991). Cellular mechanisms of fatigue in skeletal muscle. Am. J. Physiol. 261, C195–C209. 10.1152/ajpcell.1991.261.4.1-a 1872366

[B89] Wieland O. H. (1983). The mammalian pyruvate dehydrogenase complex: structure and regulation. Rev. Physiol. Biochem. Pharmacol. 96, 123–170. 10.1007/BFb0031008 6338572

[B90] Wu C. A. Chao Y. Shiah S. G. Lin W. W. (2013). Nutrient deprivation induces the Warburg effect through ROS/AMPK-dependent activation of pyruvate dehydrogenase kinase. Biochim. Biophys. Acta 1833, 1147–1156. 10.1016/j.bbamcr.2013.01.025 23376776

[B91] Xu L. Eu J. P. Meissner G. Stamler J. S. (1998). Activation of the cardiac calcium release channel (ryanodine receptor) by poly-S-nitrosylation. Science 279, 234–237. 10.1126/science.279.5348.234 9422697

[B92] Yeaman S. J. Hutcheson E. T. Roche T. E. Pettit F. H. Brown J. R. Reed L. J.. (1978). Sites of phosphorylation on pyruvate dehydrogenase from bovine kidney and heart. Biochemistry 17, 2364–2370. 10.1021/bi00605a017 678513

